# How to get the most from microarray data: advice from reverse genomics

**DOI:** 10.1186/1471-2164-15-223

**Published:** 2014-03-21

**Authors:** Ivan P Gorlov, Ji-Yeon Yang, Jinyoung Byun, Christopher Logothetis, Olga Y Gorlova, Kim-Anh Do, Christopher Amos

**Affiliations:** 1 Department of Genitourinary Medical Oncology, Unit 1374, The University of Texas MD Anderson Cancer Center, 1155 Pressler Street, Houston, TX 77030-3721, USA; 2 Department of Bioinformatics and Computational Biology, The University of Texas MD Anderson Cancer Center, Houston, TX, USA; 3 Department of Community and Family Medicine, Geisel School of Medicine, Dartmouth College, Lebanon, NH, USA; 4 Department of Eidemiology, The University of Texas MD Anderson Cancer Center, Houston, TX, USA; 5 Department of Biostatistics, The University of Texas MD Anderson Cancer Center, Houston, TX, USA

**Keywords:** Gene expression, Cancer genes, Interindividual variation in gene expression

## Abstract

**Background:**

Whole-genome profiling of gene expression is a powerful tool for identifying cancer-associated genes. Genes differentially expressed between normal and tumorous tissues are usually considered to be cancer associated. We recently demonstrated that the analysis of interindividual variation in gene expression can be useful for identifying cancer associated genes. The goal of this study was to identify the best microarray data–derived predictor of known cancer associated genes.

**Results:**

We found that the traditional approach of identifying cancer genes—identifying differentially expressed genes—is not very efficient. The analysis of interindividual variation of gene expression in tumor samples identifies cancer-associated genes more effectively. The results were consistent across 4 major types of cancer: breast, colorectal, lung, and prostate. We used recently reported cancer-associated genes (2011–2012) for validation and found that novel cancer-associated genes can be best identified by elevated variance of the gene expression in tumor samples.

**Conclusions:**

The observation that the high interindividual variation of gene expression in tumor tissues is the best predictor of cancer-associated genes is likely a result of tumor heterogeneity on gene level. Computer simulation demonstrates that in the case of heterogeneity, an assessment of variance in tumors provides a better identification of cancer genes than does the comparison of the expression in normal and tumor tissues. Our results thus challenge the current paradigm that comparing the mean expression between normal and tumorous tissues is the best approach to identifying cancer-associated genes; we found that the high interindividual variation in expression is a better approach, and that using variation would improve our chances of identifying cancer-associated genes.

## Background

Global profiling of gene expression by microarray technology is widely used to study molecular mechanisms of cancer. Even though a number of more sophisticated methods have been developed [1,2] a typical approach to analyze gene expression data is to compare the expression level between normal and primary tumor tissues [3-6]; the genes showing the largest differences in expression are usually considered to be the top candidates as cancer genes.

Recently we hypothesized that high inter-tumor variation in gene expression may more effectively identify cancer-associated genes [7]. Tumors are heterogeneous at the molecular level: in different tumors, different subsets of cancer genes are drivers and therefore are upregulated or downregulated. This leads to a higher inter-tumor variation of the drivers and only slight differences in mean expression values between normal and tumorous tissues. The goal of this study was to comprehensively evaluate that hypothesis.

A previous census of cancer-associated genes identified 400 human cancer genes [8]. The real number of cancer-associated genes is likely to be higher because the cited studies used presence of recurrent somatic mutations in tumor tissue as the only criteria to define cancer-associated genes. We can use known cancer-associated genes to identify which microarray data–derived variables are the best predictors of known cancer genes. We call this a “reverse genomics approach”, and we used it to identify the best predictors of cancer genes for the 4 most common cancers: breast, colorectal, lung, and prostate.

## Methods

### Datasets and data processing

**Figure 1:**
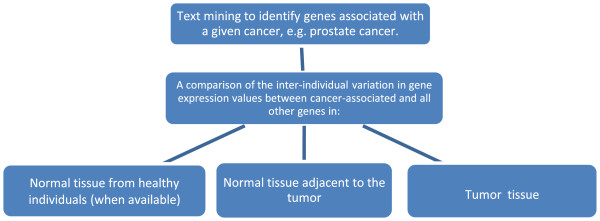
An outline of the study design.

Figure 1 outlines the design of the study. Table 1 describes the datasets we used. We applied 2 criteria for selecting the datasets: the sample size should be large enough to allow reliable estimates of interindividual variance, and the gene expression data for both tumor and adjacent normal tissues should be available. We used only those probes that could be linked to a single gene. Because different datasets used different gene identifiers, we converted them to Entrez gene identification numbers by using bioDBnet [9]. GeWorkbench 2.3.0 was used to download Simple Omnibus Format in Text files [10]; the data were log2 transformed and normalized by variance-stabilizing algorithm [11] using VCN package in R from bioconductor http://bioconductor.org/.

**Table 1:** Brief description of the datasets used

**Cancer**	**Reference no.**	**GSE_ID**	**Platform**	No. of probes*	**Sample size**	
					**AN**	**T**
Breast	[12]	GSE10780	Affymetrix HG-U133_Plus_2	35764	142	42
Breast	[13]	GDS3716	Affymetrix HuEx-1_0-st	21169	24	18
Colorectal	[14]	GSE31737	Affymetrix HG-U133_Plus_2	17528	40	40
Lung	[15]	GSE19188	Affymetrix HG-U133_Plus_2	38597	65	91
Lung	[16]	GSE18842	Affymetrix HG-U133A	38578	45	45
Prostate	[17]	GSE21034	Affymetrix HuEx-1_0-st	27090	29	29
Prostate	[18]	GSE6919	Affymetrix HG_U95Av2	27964	63	63

*Only probes linked to a single gene were used in the analysis.

AN, adjacent normal tissue; T, tumor tissue.

### Identification of known cancer genes

Our working hypothesis was that inter-individual variance for gene expression values are higher for the genes associated with cancer development because in different tumors different sets of genes are used to drive cancer development. To test this hypothesis we needed to identify genes known to be associated with a given type of cancer. We used text mining tools to identify cancer-associated genes. We evaluated 3 text mining tools: (1) Pathway Studio, (2) Ingenuity Systems, and (3) KnowledgeNet. Results were consistent across the 3 algorithms. Throughout this paper we use Pathway Studio [19] because it uses the most updated databases. The lists of known cancer-associated genes for the 4 cancers we analyzed can be found in Additional file 1. We excluded genes recently reported to be associated with cancer (defined as those reported from January 1, 2011, through July 25, 2012, the date we completed our analysis) because we used recently reported cancer genes for validation.

### Recently reported cancer genes

Our approach to retrieving recently reported cancer genes is exemplified by breast cancer genes. First we retrieved articles on breast cancer published during the period we defined as recent (from January 1, 2011, through July 25, 2012). In total, 23,550 papers were retrieved by using PubMed. We then scanned the abstracts by using MedScan [20]. One hundred forty-six associations between genes and breast cancer were identified. Seventy genes were novel and not previously reported (Additional file 2); 13 of them were microRNAs.

The same approach was used to retrieve novel colorectal, lung, and prostate cancer genes. The list of the recently reported cancer genes and the corresponding publications is found in Additional file 2.

### Microarray data–derived predictors of known cancer-associated genes

We used 6 microarray data–derived predictors: (1) mean gene expression in adjacent normal tissue (m(AN)); (2) mean gene expression in tumor tissue, (m(T)); (3) the degree of change (fold change, or FC) in expression level between tumorous and normal tissue; (4) –LOG(P), in which P is the type-I error identified by using Student’s *t* test to compare the mean expression between adjacent normal and tumor tissues; (5) the standard deviation of the gene expression in adjacent normal tissue (SD(AN)); and (6) the standard deviation of the gene expression in tumor tissue (SD(T)). Nonparametric Mann-Whitney U (MW) test was used to compare those 6 predictors between cancer-associated genes and all other genes in the human genome.

To estimate how efficiently microarray data–derived predictors predict cancer genes, we ranked the probes by the predictors’ values and estimated the percentage of the cancer-associated genes among the top 5% of the probes. An enrichment factor (EF) was used as a measure of identification efficacy: EF = P/0.05, in which P is the proportion of known cancer genes among the top 5% genes ranked by a given predictor. When EF equals 1, the predictors provide no advantage over random selection of cancer-associated genes; the higher the EF, the better the identification efficacy.

### Analysis of outliers

We then identified gene expression outliers in tumor samples separately for cancer and noncancer genes. Outliers for a given gene were defined as tumors with expression level <$\hat{m\left(N\right)}-4*\mathrm{SD}\left(N\right)$ or >$\hat{m\left(N\right)}+4*\mathrm{SD}\left(N\right)$, in which $\hat{m\left(N\right)}$ is the mean expression and *SD*(*N*) is the standard deviation of the gene expression in adjacent normal tissue. Known cancer genes are more likely to be differently expressed and therefore are more likely to be outliers. To account for the effect of differential gene expression on its probability to be an outlier we first i) sorted all genes according to –LOG(P) from largest to smallest, and for each known cancer gene, and then ii) took non-cancer genes from the list immediately above and below of a given known cancer genes. Those neighbouring non-cancer genes were used as a comparison group.

### Computer simulation

We used computer simulation to compare the efficacy of identification of cancer genes by SD(T) and –LOG(P). We simulated the expression levels of 1,000 genes: 50 cancer genes and 950 noncancer genes. The total sample size was 40 tissues: 20 adjacent normal tissues and 20 tumor tissues. Expression values were sampled from the normal distribution with mean = 7 and *SD* = 0.6 {*N*(7.0,0.6)}, which are typical means and *SD*s for the datasets we used.

**Figure 2:**
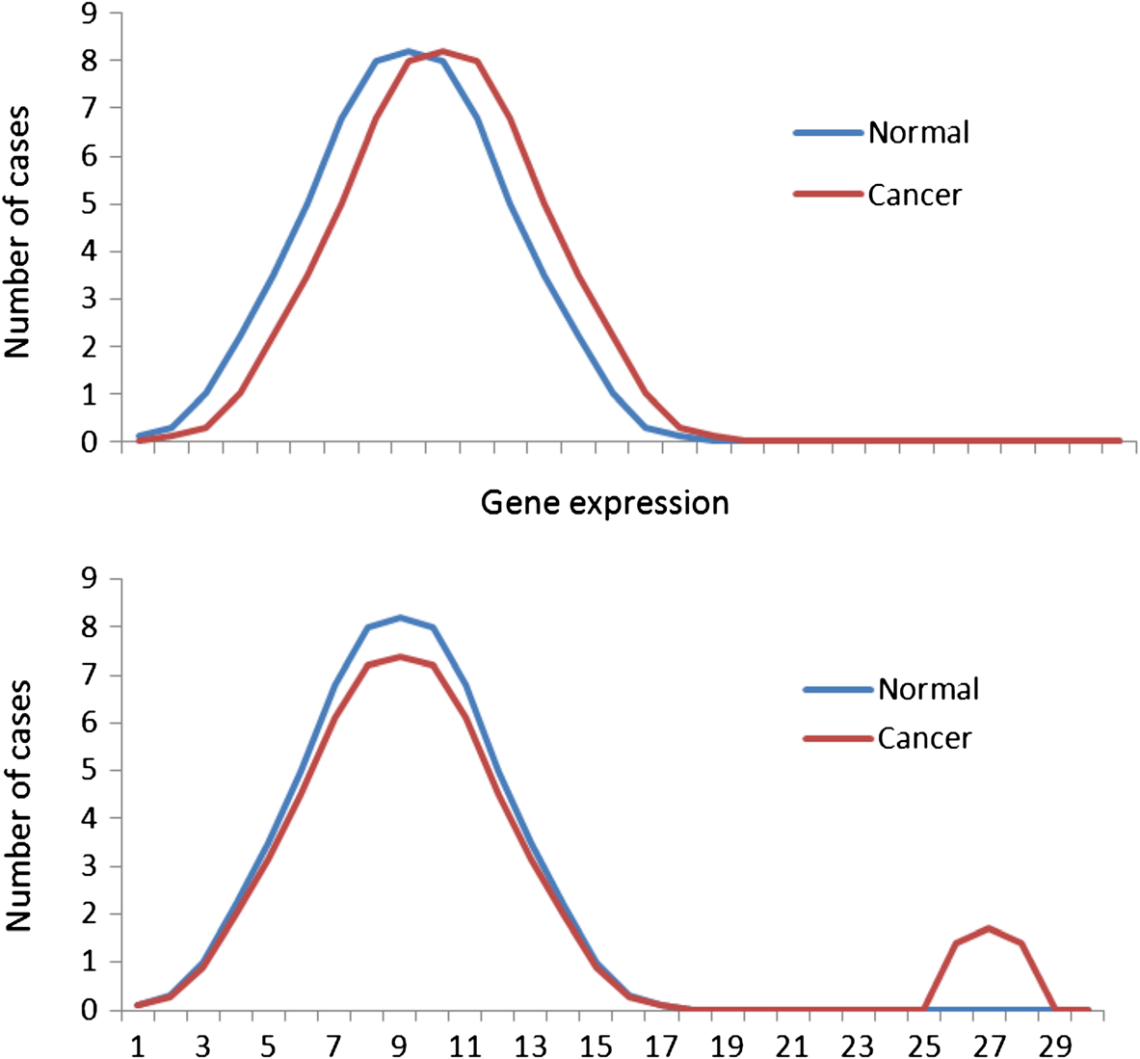
**The “shifting means model” (upper panel) and the “outliers model” (lower panel) of gene expression in tumors.** In the shifting means model, all tumors are similar in terms of gene expression. In the outlier’s model, the tumors are heterogeneous: a specific cancer gene is extremely upregulated or downregulated only in a small fraction of tumors in which this gene is a driver of tumorigenesis.

Two models were compared: the “shifting means model” and the “outlier’s model” (Figure 2). In the shifting means model, tumors are homogeneous: differences in mean expression levels between normal and tumor tissues are due to the shift of the distribution to the right (upregulation) or the left (downregulation). In the outlier’s scenario, a cancer gene is differently expressed in only a fraction of tumors. In different tumors, different cancer genes can be outliers.

In the shifting means model, expression values were sampled from the normal distribution: *N*(7.0 + *s*, 0.6), in which *s* is the difference in mean expression values between tumor and adjacent normal tissues. Those mean and variance are typical for the studies we have used. Three different values of *s* (0.07, 0.35, and 0.7) were considered. The number of simulated outliers was defined to make the mean expression value in tumors similar to that of the shifting means model.

### Binary logistic regression model

To explore whether the combination of several predictors can provide better identification of known cancer genes than any single predictor can, we ran a binary logistic regression model. A stepwise-forward likelihood ratio model was used to identify significant predictors in each dataset. This analysis was done for each cancer type separately. Known cancer genes were considered as outcome and –LOG(P), m(AN), m(T), FC, SD(AN), and SD(T) as predictors.

### Raw and processed data

To estimate the effect of the data processing on the variance estimates, we computed variance in adjacent normal and tumor samples of (1) raw gene expression data, (2) log(2)-transformed data, and (3) data normalized by using a variance-stabilizing approach. We compared variance through these 3 levels using Kendall’s rank-correlation and estimated the efficacy of the variance-based identification of cancer genes for each level.

## Results

### SD(T) shows the strongest association with known cancer genes

The lists of the known breast, colorectal, lung, and prostate cancer genes are shown in Additional file 2. To check how known cancer genes differ from other genes, we used nonparametric MW testing; Table 2 shows the results of the comparison. SD(T) consistently revealed the strongest differences between cancer and noncancer genes: in 6 of 7 datasets, SD(T) had the largest MW statistics.

**Table 2:** Differences between cancer and all other genes for 4 cancer gene predictors in 7 datasets

**Dataset**	**Trait**	**Mean CG**	**Mean OG**	**Z**	**P value**	**Rank**
BC_GSE10780	-LOG(P)	6.09	5.84	2.89	0.003852	6
	m(AN)	6.32	6.16	3.68	0.000233	4
	m(T)	6.51	6.18	3.3	0.000967	5
	FC	1.33	1.19	4.65	3.32E-06	3
	sd(AN)	0.42	0.33	5.61	2.02E-08	1
	sd(T)	0.62	0.45	5.37	7.87E-08	2
BC_GSE3716	-LOG(P)	0.62	0.64	1.04	0.29834	3
	m(AN)	7.34	7.42	0.37	0.711382	6
	m(T)	7.41	7.51	0.75	0.453255	4
	FC	1.21	1.21	0.58	0.561915	5
	sd(AN)	0.84	0.79	2.79	0.005271	2
	sd(T)	0.89	0.81	3.55	0.000385	1
CC_GSE31737	-LOG(P)	3.51	2.53	4.64	3.48E-06	6
	m(AN)	5.74	4.77	5.54	3.02E-08	5
	m(T)	5.89	4.77	6.19	6.02E-10	4
	FC	1.40	1.19	6.58	4.7E-11	2
	sd(AN)	0.38	0.22	6.52	7.03E-11	3
	sd(T)	0.56	0.24	7.53	5.07E-14	1
LC_GSE19188	-LOG(P)	6.55	5.37	1.15	0.250144	6
	m(AN)	6.49	6.06	4.74	2.14E-06	4
	m(T)	6.61	6.12	4,47	7.82E-06	5
	FC	1.58	1.29	5.62	1.91E-08	3
	sd(AN)	0.34	0.17	7.86	3.84E-15	2
	sd(T)	0.93	0.56	10.77	4.77E-27	1
LC_GSE18842	-LOG(P)	5.98	4.31	3.08	0.00207	6
	m(AN)	6.24	5.75	6.26	3.85E-10	4
	m(T)	6.47	5.75	6.56	5.38E-11	3
	FC	1.66	1.31	6.1	1.06E-09	5
	sd(AN)	0.43	0.33	8.21	2.21E-16	2
	sd(T)	0.75	0.47	8.72	2.78E-18	1
PC_GSE6919	-LOG(P)	2.28	1.75	3.62	0.000295	4
	m(AN)	7.41	7.01	1.87	0.061484	5
	m(T)	7.42	6.99	1.86	0.062886	6
	FC	1.32	1.21	4,57	4.88E-06	2
	sd(AN)	0.61	0.59	3.87	0.000109	3
	sd(T)	0.74	0.64	4.71	2.48E-06	1
PC_GSE21034	-LOG(P)	2.55	1.68	3.97	7.19E-05	6
	m(AN)	8.65	7.86	6.82	9.1E-12	4
	m(T)	8.59	7.82	5.87	4.36E-09	5
	FC	1.21	1.12	6.78	1.2E-11	3
	sd(AN)	0.31	0.27	6.3	2.98E-10	3
	sd(T)	0.37	0.28	6.83	8.49E-12	1

CG, cancer genes; OG, other genes. Statistics from nonparametric Mann-Whitney test; rank, rank of the variable for a given dataset based on *Z* score; m(AN), mean expression in adjacent normal tissue: m(T), mean expression in tumor tissue; FC, fold change; SD(AN), standard deviation of the gene expression values in adjacent normal tissue; SD(T), standard deviation of the gene expression values in tumor tissue.

**Figure 3:**
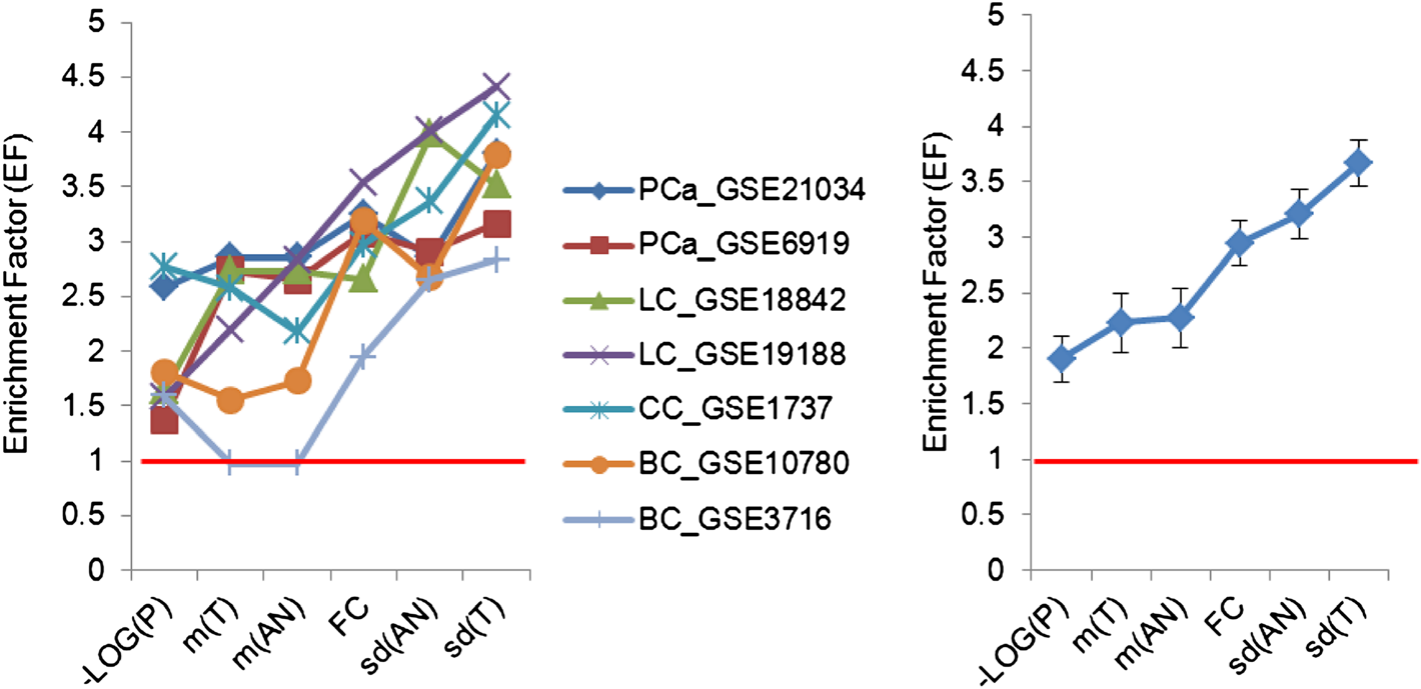
**The enrichment factor (EF) for known cancer genes among the top 5% of the probes ranked on the basis of the predicting variables.** The horizontal lines show the expected proportion of cancer genes under the null hypothesis. Left panel shows individual studies, right panel shows averages across the studies.

We also ranked the probes according to the predicting variables and estimated percentage of the known cancer genes among the top 5% of the probes. Under the null hypothesis, one can expect 5% of the known cancer genes to be among the top 5% of the ranked probes. We found that all predictors identified more known cancer genes than what one could expect by chance. Figure 3 shows the results of that analysis.

### Outliers

In all datasets, the percentage of outliers for cancer genes was higher than it was for the comparison group (Table 3). In 3 datasets, the differences were significant. In the overall analysis (nonparametric MW test), the percentage of outliers was overall higher for cancer genes than it was for the paired controls: 3.3 ± 0.2 vs. 2.6 ± 0.1; MW test *Z* = 3.5; *P* = 0.0004.

**Table 3:** Percentage of outliers in tumor samples

**Dataset**	**Probe Type**	**% of Outliers**			***Z***	***P***
		**Mean**	**N**	**SE**		
GSE3716_BC	Paired controls	7.01	439	0.38		
	Cancer genes	7.65	226	0.54	1.08	0.22
GSE10780_BC	Paired controls	4.36	457	0.38		
	Cancer genes	4.59	469	0.41	0.84	0.61
GSE31737_CC	Paired controls	**1.26**	**202**	**0.18**		
	Cancer genes	**2.78**	**101**	**0.59**	**2.28**	**0.005**
GSE18842_LC	Paired controls	**9.47**	**506**	**0.84**		
	Cancer genes	**12.68**	**256**	**1.40**	**2.26**	**0.01**
GSE19188_LC	Paired controls	**6.97**	**506**	**0.55**		
	Cancer genes	**10.89**	**254**	**0.98**	**2.12**	**0.03**
GSE6919_PC	Paired controls	0.81	457	0.08		
	Cancer genes	0.98	230	0.11	0.56	0.74
GSE21034_PC	Paired controls	1.72	343	0.34		
	Cancer genes	2.51	182	0.52	1.03	0.11

Data in bold face are statistically significant between paired controls and cancer genes.

### Recently reported cancer-associated genes

We next compared the newly reported cancer genes (i.e., reported from January 1, 2011, through July 25, 2012) with all other genes in the human genome (Table 4). SD(T) was the most significant variable for recently identified cancer genes.

**Table 4:** Differences between recently reported cancer genes and all other genes in the human genome

**Dataset**	**Trait**	**Mean RRCGs**	**Mean OGs**	***Z***	***P *** **value**	**Rank**
BC_GSE10780	-LOG(P)	5.81	4.36	2.33	0.019828	6
	m(AN)	6.65	6.16	3.09	0.002	5
	m(T)	6.77	6.18	3.61	0.0003	3
	FC	0.37	0.25	3.43081	0.000602	4
	sd(AN)	0.31	0.41	3.88355	0.000103	2
	sd(T)	0.62	0.45	4.52874	0.000006	1
BC_GSE3716	-LOG(P)	0.53	0.64	1.27891	0.200931	2
	m(AN)	7.74	7.43	0.92	0.36	4
	m(T)	7.79	7.50	0.79	0.43	5
	FC	0.26	0.27	0.26448	0.791411	6
	sd(AN)	0.82	0.79	1.2498	0.211373	3
	sd(T)	0.89	0.80	2.71994	0.00653	1
CC_GSE31737	-LOG(P)	2.99	2.38	1.41345	0.157523	6
	m(AN)	5.24	4.78	2.11	0.04	4
	m(T)	5.24	4.78	2.06	0.04	5
	FC	0.40	0.25	2.78341	0.005379	3
	sd(AN)	0.52	0.40	3.52082	0.00043	2
	sd(T)	0.57	0.43	4.12776	0.000037	1
LC_GSE19188	-LOG(P)	6.47	5.38	1.91017	0.056112	6
	m(AN)	6.55	6.24	2.01	0.04	5
	m(T)	6.71	6.57	2.11	0.04	4
	FC	0.61	0.38	4.50134	0.000007	3
	sd(AN)	0.52	0.35	5.09778	<10-6	2
	sd(T)	0.84	0.56	7.51503	<10-6	1
LC_GSE18842	-LOG(P)	6.01	4.32	3.59092	0.00033	6
	m(AN)	6.67	5.75	3.71	0.0002	5
	m(T)	6.81	5.75	4.91	0.000001	4
	FC	0.78	0.39	5.99594	<10-6	2
	sd(AN)	0.51	0.33	5.1629	<10-6	3
	sd(T)	0.82	0.47	8.26221	<10-6	1
PC_GSE6919	-LOG(P)	0.94	0.87	0.74248	0.457796	6
	m(AN)	7.16	7.01	0.92	0.36	5
	m(T)	7.18	6.99	1.04	0.3	4
	FC	0.17	0.14	1.47057	0.141409	3
	sd(AN)	0.63	0.59	1.84723	0.064715	2
	sd(T)	0.69	0.64	2.15976	0.030792	1
PC_GSE21034	-LOG(P)	1.69	1.69	0.74953	0.453541	6
	m(AN)	8.09	7.87	2.16	0.03	4
	m(T)	8.15	7.83	2.25	0.02	3
	FC	0.24	0.16	1.98956	0.04664	5
	sd(AN)	0.32	0.27	2.66829	0.007624	2
	sd(T)	0.34	0.29	2.99909	0.002708	1

RRCG, recently reported cancer genes; OG, other genes.

### Computer simulation

**Figure 4:**
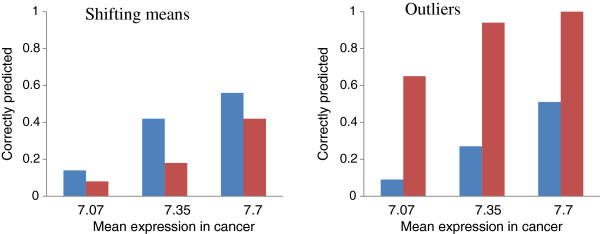
**The proportions of correctly predicted cancer genes for the shifting means (left panel) and outliers (right panel) models.** The prediction based on – LOG(P) is shown in blue, and that based on SD(T) is shown in red.

Figure 4 illustrates the results of the computer simulation. We found that for the shifting means model, –LOG(P) performed better than SD(T) did; however, for the outliers model, the identification efficacy was better for SD(T).

### Binary logistic regression model

The results of applying the binary logistic regression model to individual datasets are shown in Table 5. We found that SD(T) was the most significant predictor of known cancer genes in all models; in 3 of the 7 models, SD(T) was the single significant predictor. For the other predictors, the results were inconsistent across studies.

**Table 5:** Results of applying the binary logistic regression model to the 7 datasets

**Cancer**	**Dataset**			**Variables in the model**			
		**–LOG(P)**	**m(AN)**	**M(T)**	**FC**	**SD(AN)**	**SD(T)**
Breast	GDS3716	ns	Ns	Ns	ns	Ns	10.5(0.001)
Breast	GSE10780	ns	Ns	Ns	ns	Ns	76.1(<10-6)
Colorectal	GSE31737	5.2(0.02)	Ns	19.6(<10-6)1.5 E-82	ns	Ns	27.8(<10-6)
Lung	GSE18842	ns	Ns	13.1(<10-6)3.3 E-39	15.2(3.5 E-52)	6.5(0.01)	41.5(<10-6)
Lung	GSE19188	ns	Ns	Ns	ns	Ns	220.1(<10-6)
Prostate	GSE6919	7.9(0.005)		7.1(0.007)	ns	Ns	74.9(<10-6)
Prostate	GSE21034	22.3(<10-6)		4.8(0.04)	18.9	8.1(0.004)	48.8(<10-6)

ns – the variable is not significant; numbers are Wald statistics for the variables in the model; significance is shown in parentheses.

### Raw and processed data

We found a strong correlation between variances of log(2)-transformed and normalized data: Kendall’s rank-correlation coefficient varied from 0.94 to 0.98. The prediction efficacy was essentially the same for the 2 types of data. For the raw data, SD(T) was the most significant predictor of known PCa genes. The average EF was slightly lower for the raw data than was it was for the processed data: 3.5 ± 0.3 vs. 3.8 ± 0.2, even though the correlation between the raw-data variance and the processed-data variance was relatively low: average, 0.52; range, 0.31 to 0.62.

## Discussion

Our study showed that assessment of the interindividual variation of gene expression is better at predicting cancer-associated genes than is the traditional comparison of mean gene expression in adjacent normal and tumor tissues. The results were consistent for 4 major cancer types: breast, colorectal, lung, and prostate cancer. Additionally, we checked bladder cancer data (dataset GDS1479) [21] and found that SD(T) was also the best predictor of known bladder cancer genes (data not shown).

Why does interindividual variation in gene expression provide a more effective approach to identifying cancer genes than do differences in the mean expression? We believe that tumor heterogeneity at the genetic level is the most likely reason. Although technical errors can contribute to interindividual heterogeneity of gene expression, their effect should not be specific to cancer genes and usually it is much lower than that of gene expression heterogeneity resulting from biologic differences in gene expression [22-24].

It is well recognized that different tumors are driven by different sets of genes [25-28]. Variation among tumors will lead to a substantial increase in interindividual variation of cancer-associated genes and only slight differences in means. The results of our computer simulation support this explanation. When differences in expression levels between normal and tumor tissues are driven by outliers, SD(T) provides a superior prediction of cancer genes than –LOG(P) does. Consistent with these results, we found that cancer genes have a higher probability of being outliers than do other genes with comparable levels of differential expression between tumorous and adjacent normal tissue.

SD-based prediction of cancer genes seems to be robust for data processing. Regardless of whether raw or processed data were used for predicting cancer genes, the average enrichment factor was highest for SD(T). This is likely related to the fact that in extreme cases (i.e., very low or very high variance), it does not really matter whether we are using raw or processed data because the most variable genes tend to keep the same ranking across the different levels of data processing.

The binary logistic regression model identified SD(T) as the most significant and often the only predictor of known cancer genes, whereas the results for the other predictors we tested were inconsistent. Thus, our preliminary analysis does not support the idea that combining several predictors could be better than the cancer genes identification based on SD(T) only.

We noted that interindividual variation in the expression levels of cancer genes was higher not only for tumor samples but also for adjacent normal tissues. This may be a result of selection: adjacent normal tissue is not the same as normal tissue from healthy individuals. People differ by expression levels of cancer genes in normal target tissue, and those differences can contribute to the risk of developing cancer. Somatic alterations, such as methylation or loss or gain of chromosomal regions, may further modulate the expression of cancer genes [29-34], which may explain the better prediction of cancer genes by SD(T) than by SD(AN). The best way to validate this selection hypothesis would be to compare the gene expression in normal tissue (i.e., free of any pathologic changes) with that in adjacent normal tissues from cancer patients. Unfortunately, data on gene expression in normal tissues are usually not available. The only available dataset we found was GSE6919. For that dataset, we found that in normal prostate tissue from healthy individuals, the mean SD for cancer genes was 0.63 ± 0.03, and the mean SD for all other genes was 0.61 ± 0.01. That difference was not statistically significant (MW test, *Z* = 1.01, *P* = 0.31). However, in “normal” tissue adjacent to tumor, the genes associated with prostate cancer showed larger interindividual variation in expression than all other genes have: MW test, Z = 4.57, *P* = 0.000005. This suggests that “normal” tissue from cancer patients is different from normal prostate tissue from healthy individuals. It also suggests that the population of prostate cancer patients is heterogeneous in terms of the expression of prostate cancer genes: different sets of prostate cancer genes are upregulated or downregulated in different patients, leading to a greater interindividual variation in expression.

If a higher interindividual variation in the expression of cancer-associated genes results from genetic heterogeneity, so that different tumors use different sets of cancer genes, one can expect that genetically homogeneous cancers would not show high interindividual variation in expression. Clear cell renal cell carcinoma (CCRCC) is believed to be one of the least heterogeneous cancers [35] with only two major subtypes (ccA and ccB) identified by expression profiling [36]. For CCRCC we used GSE781 GEO dataset [37]. This dataset was selected because it was generated using Affymetrix U133A platform which makes the results comparable with the results on breast, colorectal and lung cancers.

Interindividual variation in expression values of CCRCC-associated genes was not different from interindividual variation in expression values for all other genes, neither in tumor: 0.24 ± 0.01 vs 0.23 ± 0.01; Mann-Whitney U Test Z-adjusted = 0.7, P = 0.48, nor in adjacent normal tissues: 0.23 ± 0.01 vs 0.22 ± 0.01; Mann-Whitney U Test Z-adjusted = 0.3, P = 0.74. Therefore the results of this analysis support the idea that a lower level of interindividual variation in the expression of cancer-associated genes is a result of tumor genetic homogeneity in this particular cancer type. However, in this specific analysis the lack of difference can be due to smaller sample size (9 tumor and 9 adjacent normal samples). This sample size is much smaller compared to the sample sizes used for other types of cancer (Table 1). To address this issue we randomly sampled 9 tumors from each of the datasets used for the analysis of the other types of cancer (Table 1). Twenty random samplings were performed for each datasets, 140 runs in total. For 134 of them or 96%, SD for cancer-associated genes was significantly higher compared to SD for all other genes. This suggests that a smaller sample size is unlikely to explain the lack of differences in SD between cancer-associated and all other genes in CCRCC sample. Therefore, the results of the analysis of CCRCC support the idea that genetic heterogeneity contributes to the higher interindividual variation in the expression of cancer-associated genes, but a larger study is needed to be definitive on this point.

## Conclusion

In conclusion, we found that interindividual variation in gene expression more effectively identifies known cancer genes than does the difference in mean expression levels between adjacent normal and tumor tissues. The variation in gene expression levels was more effective at identifying know cancer genes than were differences in mean levels or p-values. Thus, if we use SD(T) instead of the traditional –LOG(P), we would increase our chances of identifying cancer-associated genes. Overall, our results suggest that it would be beneficial to analyze interindividual variation in gene expression.

## Abbreviations

EF: Enrichment factor; FC: Fold change in gene expression between tumorous and normal tissue; –LOG(P): P is the type-I error from Student’s *t* test, comparing mean expression in adjacent normal and tumor tissues; m(AN): Mean gene expression in adjacent normal tissue; m(T): Mean gene expression in tumor tissue; MW: Mann-Whitney U testing; SD(AN): Standard deviation of the gene expression in adjacent normal tissue; SD(T): Standard deviation of the gene expression in tumor tissue.

## Competing interests

The authors declare that they have no competing interests.

## Authors’ contributions

IG conceived the study and drafted the manuscript. JY participated in the bioinformatics and statistical analysis, JB participated in the statistical analysis, CL helped draft the manuscript, OG participated in the design of the study and helped to draft the manuscript, KD participated in the design of the study, CA participated in the design of the study and analyses. All authors read and approved the final manuscript.

## Supplementary Material

Additional file 1 Known cancer genes. Click here for file

Additional file 2 Recently identified cancer genes. Click here for file
